# Induction of Stress Granules and Developmental Instability of Offspring Phenotype Due to Hypothermia During First Mouse Embryo Cleavage

**DOI:** 10.3390/ijms26168060

**Published:** 2025-08-20

**Authors:** Galina Kontsevaya, Alexander Romashchenko, Tatyana Babochkina, Dasha Sugatova, Oleg Shevelev, Marina Sharapova, Yuri Moshkin, Mikhail Moshkin, Ludmila Gerlinskaya

**Affiliations:** 1 Federal Research Center Institute of Cytology and Genetics, Siberian Branch of RAS, Lavrentyeva 10, 630090 Novosibirsk, Russia; koncevayagalina@bionet.nsc.ru (G.K.); babochkinat@bionet.nsc.ru (T.B.); dasha.sugatova@mail.ru (D.S.); shevelev@bionet.nsc.ru (O.S.); sharapova@bionet.nsc.ru (M.S.); moshkin.yuri@gmail.com (Y.M.); 2 LIFT Center LLC, 121205 Moscow, Russia; 3 Gene Learning Association, 1205 Geneva, Switzerland; 4 Department of Vertebrate Zoology and Ecology, Tomsk State University, 634050 Tomsk, Russia

**Keywords:** preimplantation embryo, temperature, stress granules, embryo viability, offspring, gene “noise”, hippocampus

## Abstract

Zygotic genome activation (ZGA) represents one of the most vulnerable periods to environmental perturbations. The objective of this study was to investigate the formation of stress granules in mouse embryos in response to temperature reduction during ZGA, preimplantation embryo mortality, and long-term phenotypic outcomes. These outcomes included the evaluation of expression noise in bilateral right/left limbs of offspring as an indicator of developmental instability, behavioral deviation, hippocampal volume, and metabolomics profiling in adult offspring. Exposure to hypothermia during ZGA was associated with an increased number and inter-blastomere variability of stress granules, extended duration of the second embryonic division, and elevated embryonic mortality during the second and third cleavage stages. The embryonic response to hypothermic stress correlated with phenotypic traits indicative of increased pathology risk. Expression noise, serving as an indicator of developmental instability, was reduced in adult offspring derived from two-cell embryos incubated at 35 °C compared to those at 37 °C, while showing no significant difference relative to the control group. These results suggest that embryos surviving hypothermic exposure (35 °C) possess enhanced resilience to the adverse effects commonly associated with embryo transfer procedures. Furthermore, increased hippocampal volume and augmented auditory startle reflex observed in offspring that endured hypothermia during ZGA imply reduced risks of cognitive-related pathologies and reduced risks of pathologies associated with cognitive functions.

## 1. Introduction

The use of assisted reproductive technologies (ART), which help overcome infertility issues, increases by 6.7% annually [1]. Nevertheless, there is growing evidence that pregnancies following ART are associated with an increased risk of perinatal complications and pathologies in offspring. However, despite the ongoing global expansion of ART, only a few studies have systematically investigated the long-term health implications associated with these procedures [2].

The preimplantation stage of pregnancy is characterized by high rates of embryonic loss. After in vitro fertilization, only 30–50% of fertilized eggs reach the blastocyst stage. The reasons for this phenomenon are not fully understood. The preimplantation period represents a critical window of vulnerability, as it is a time of global epigenetic reprogramming in embryos. Perturbations in environmental conditions capable of disrupting DNA methylation patterns during this phase may predispose offspring to adverse health outcomes. For example, thermal stress has been shown to impact metabolic processes, the intracellular matrix, the epigenetic landscape, and apoptotic pathways. Collectively, this leads to impaired embryonic development and increased embryonic mortality [3,4,5]. The loss of global DNA methylation during reprogramming initiates zygotic genome activation (ZGA), a process essential for normal embryogenesis. Investigations into the effects of temperature modulation during ZGA have demonstrated that increased intra-embryonic variability of 5mC methylation at the two- and four-cell stages is increased and subsequently normalized to in vivo levels by the eight-cell embryos incubated at 35 °C [6].

Elevated variability in 5-methylcytosine (5mC ) methylation has been associated with peak embryonic mortality during the first three cleavage divisions. The loss of embryos during this early developmental stage is a significant factor in the overall losses observed during subsequent gestation and neonatal stages [6]. This embryonic lethality may be attributable to developmental instability (DI), as evidenced by increased intra-embryonic heterogeneity in blastomere size at the two- and four-cell stages, alongside fluctuations in 5mC levels within blastomere nuclei, which decrease in viable eight-cell embryos to the level seen in those developing in vivo. However, the long-term consequences of severe low-temperature exposure, as evidenced by early embryonic lethality, on the phenotype of sexually mature offspring remain unclear. It is well-known that cells employ a variety of adaptive pathways to respond to various types of stress, including temperature variations. There are specific mechanisms that allow cells to compensate for changes in temperature, pH, oxygen tension, nutrient availability, and other environmental factors. The cellular response to stressful influences is directly linked to the regulation of protein synthesis, transport, and post-translational modifications, as well as overall cell viability [7]. One of the earliest intracellular responses to adverse external conditions is the formation of temporary, membrane-less stress granules (SG) in the cytoplasm. The main function of SG is to accumulate untranslated mRNAs and suppress protein synthesis at the ribosomal level [8,9]. Thus, SG minimizes cellular energy expenditure, controls protein homeostasis, and regulates RNA metabolism [10,11]. Stress granules are thought to play a protective role during stress conditions, but their exact biological function remains unclear. In general, the question of the long-term consequences of brief extreme exposures during critical periods of preimplantation development on traits associated with DI and, consequently, the increased risk of pathologies in offspring remains an open question.

The phenotypic fluctuating asymmetry (FA) is usually attributed to developmental stability or developmental instability (DI) [12,13]. In general, the organism’s development program is determined by the coordinated expression of genes. Multicellular organisms are bilaterally symmetrical, and it is expected that gene expression in left/right body structures will also be the same and that the body will have perfect symmetry, which is attributed to developmental stability [14]. The phenotypic symmetry of bilateral structures is the result of the symmetric and equally balanced process of the achievement of the genetic program in multicellular organisms. However, discrete and inherently random events are involved in the realization process of the gene expression program during all the stages of the implementation of genetic information. As a result, random stochastic fluctuations have been widely observed in the gene expression of both prokaryotic and eukaryotic cells [15,16,17,18]. These random stochastic fluctuations result in the stochastic creation of different genetic program realization patterns between genetically identical cell populations in the multicellular organism. Single-molecule studies performed at the single-cell resolution level have demonstrated that gene expression is inherently stochastic, where even amongst isogenic cells, a gene’s expression level is not identically distributed [16]. The different gene expression pattern in the bilateral structures of multicellular organisms produces the gene expression FA of traits in the adult phenotype, which depends on genomic and transcriptional stability, proteostasis, and epigenetic buffering of development [19,20].

Stability of ontogenetic processes is necessary for the formation of the phenotype without disturbances and failures. We demonstrated the dependence of FA on various MHC combinations of the surrogate mother and fetuses developed after intra- and interline transferences of two-cell embryos, as well as the positive effect of distant MHC combinations on embryonic growth [21]. According to recent data, the embryo develops in a temperature gradient from higher temperature during fertilization to lower temperature during subsequent development and movement along the reproductive tract [22,23]. Some evidence suggests that mimicking natural changes in incubation temperature has a beneficial effect on preimplantation embryo development [24,25]. Our previous studies [7] demonstrated that exposure to reduced temperature during ZGA amplifies variability in DNA methylation patterns and blastomere size. Additionally, incubation at 35 °C during the first cleavage stage resulted in a marked decrease in the proportion of embryos progressing to the third cleavage division relative to embryos maintained at the standard 37 °C temperature. These observations suggest that lowered temperature during ZGA imposes selective pressure on the developing embryos. It is still unclear how short-term adverse environmental factors at the stage of the first embryonic division affect the phenotypic stability of adult offspring.

The association between the reduction in temperature during ZGA and the expression noise in the bilateral right/left forelimbs, behavior, hippocampal volume, and metabolomic profiling of adult offspring was examined in this study. Notably, the hippocampus volume and FA of embryos that survived at low temperatures were similar to those of the control group. Our findings indicated that the surviving cohort may have adaptive characteristics that increase their resistance to the adverse conditions that are typically associated with ART pregnancies.

## 2. Results

### 2.1. Development of Early Preimplantation Embryos

**Figure 1:**
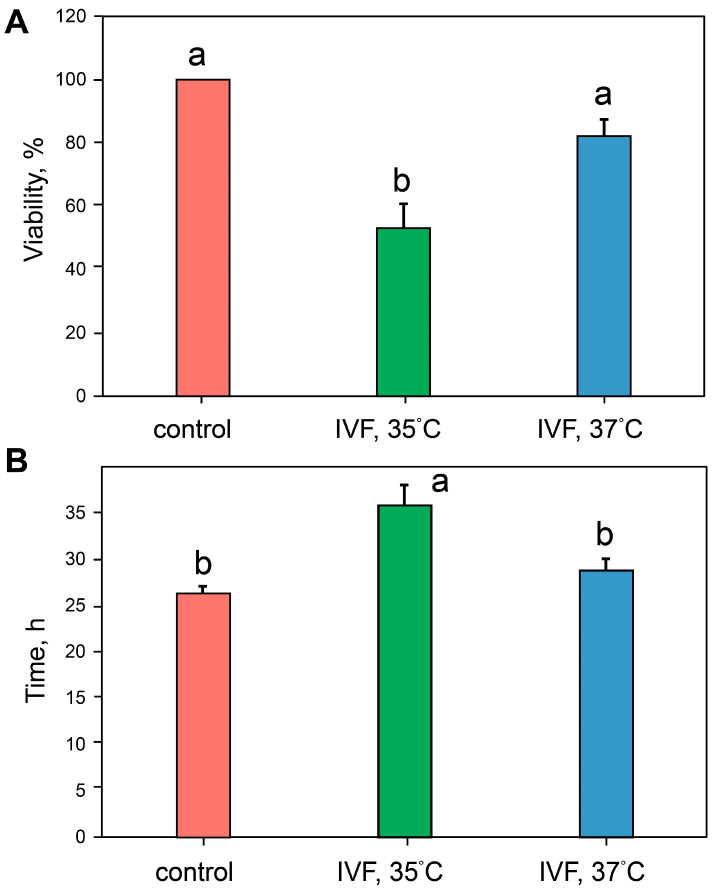
Embryo response to reduced incubation temperature. (**A**) The impact of different incubation temperatures during the first 24 h following IVF on the survival of embryos at the third division stage was assessed. All embryos in the control group reached the 8-cell stage. (**B**) Duration of the second cell division: a,b—different letters denote groups that differ significantly (LSD test, *p* < 0.05).

The temperature conditions during incubation at the first embryonic division stage significantly affected (H_2,18_ = 11.80, *p* = 0.0027, Kruskal–Wallis test) the proportion of embryos that died during the second and third embryonic divisions. Lowering the incubation temperature to 35 °C at the first division stage was associated with a significant reduction in the proportion of embryos completing the third division compared to embryos incubated constantly at 37 °C (Z = 2.29, *p* = 0.014, Mann–Whitney test) (Figure 1A). Similar differences were observed between groups of embryos obtained by natural in vivo fertilization (control) and IVF at 35 °C (Z = 2.54, *p* = 0.0095, Mann–Whitney test). The incubation temperature at the first division stage also influenced the timing of the second embryonic division (F_2,112_ = 8.98, *p* = 0.0002, ANOVA test). The duration of the second embryonic division was the longest in the IVF 35 °C group (n = 39) compared to the IVF 37 °C (n = 55) and control (n = 29) groups (Figure 1B).

Furthermore, two-way ANOVA revealed differences in second division timing between groups containing dead embryos (IVF 35 °C and IVF 37 °C). Specifically, the duration of the second division was affected by the incubation temperature factor (F1 = 5.64, *p* < 0.02) and the survival/death factor (F1 = 56.92, *p* < 0.0001). The duration of the second division was significantly longer in embryos that died compared to those that survived. In the IVF, 35 °C group, non-viable embryos exhibited a second cleavage time of 31.5 ± 1.17 h (n = 22), whereas viable embryos showed 44.6 ± 5.4 h (n = 11), t = 3.23, df = 31, *p* < 0.0000. Similarly, in the IVF 37 °C group, the values were 25.6 ± 0.70 h (n = 44) for non-viable embryos and 44.6 ± 5.4 h (n = 9) for viable embryos, t = 9.00, df = 51, *p* < 0.0001.

### 2.2. Stress Granules

**Figure 2:**
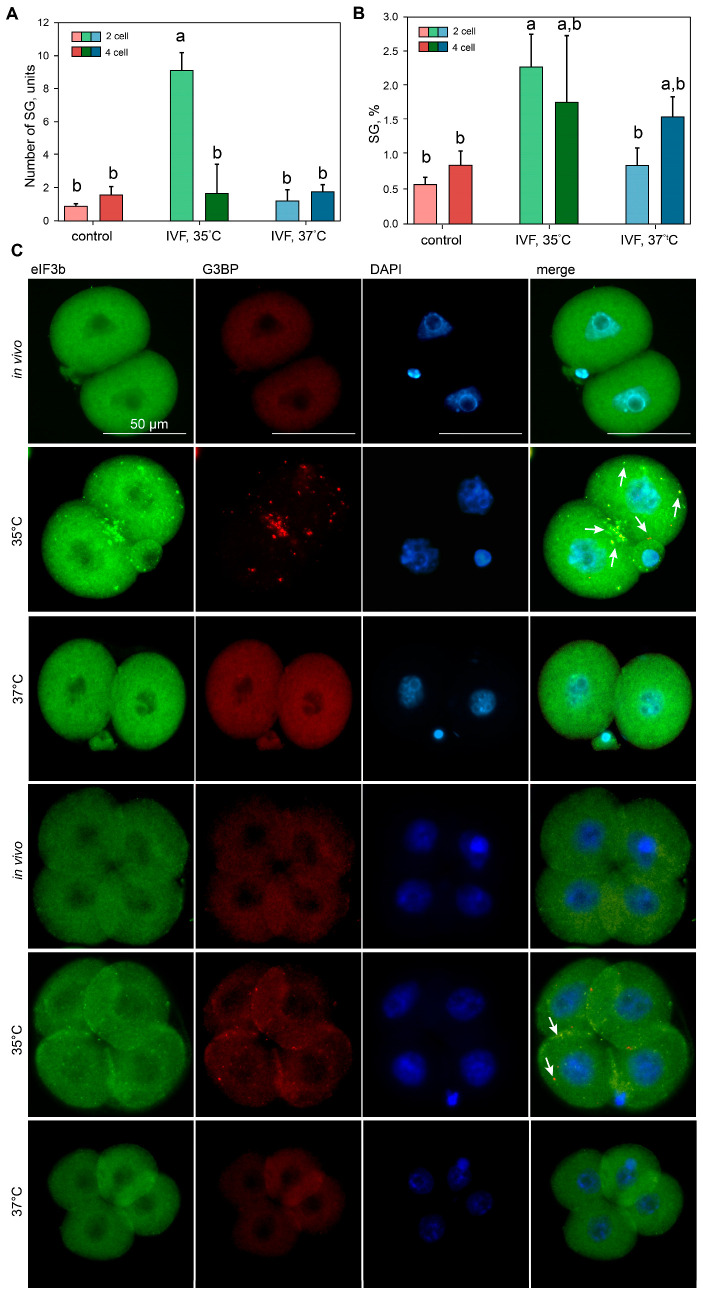
Stress granule numbers and inter-blastomere variability in response to reduced incubation temperature. (**A**) The average number of SGs per embryo; (**B**) inter-blastomere distribution of SGs. (**C**) Examples of stress granules in embryos incubated under different conditions. White arrows indicate stress granule formations. Protein aggregates were color-coded: yellow for eIF3b+G3BP complexes, red for G3BP-only assemblies. The inter-blastomere coefficient of variation of SG numbers within a single embryo was used: a,b—letters indicate groups that differ significantly (Kruskal–Wallis test *p* < 0.000) and pairwise comparisons (*p* < 0.002; Mann–Whitney test).

The development of protein agglomerates comprising stress granule-associated proteins eIF3η and G3BP1 was generated by incubating embryos at 35 °C during the first 24 h following IVF (Figure 2). A selective inhibitor of eIF2α (the α subunit of eukaryotic translation initiation factor 2) and an inhibitor of phosphorylation of PERK kinase, a crucial mediator of the unfolded protein response (UPR), both affected the assembly of these agglomerates under lower incubation temperature conditions in the IVF, 35 °C group (Figure S1A). Interestingly, these protein agglomerates were temporary; their quantities dropped to control levels following embryo transfer to 37 °C (Figure S1B). The quick dissociation of canonical stress granules following stress termination is consistent with this tendency [11]. In light of these findings, we carefully categorized the detected protein agglomerates as stress granules.

The adaptive response to incubation temperature conditions was assessed by counting stress granules in embryos at the two-cell stage: control (n = 36), IVF, 35 °C (n = 17), IVF, 37 °C (n = 10); and at the four-cell stage: control (n = 29), IVF, 35 °C (n = 6), IVF, 37 °C (n = 26). Stress granule formation during the first division was significantly influenced by the incubation temperature (H_2,63_ = 24.85, *p* < 0.000) (Figure 2A). After the completion of the first division, the number of stress granules was the highest in the IVF group at 35 °C compared to the control group (Z = 3.01, *p* < 0.002) and the IVF group at 37 °C (Z = 4.45, *p* < 0.002) (Mann–Whitney test).

The number of stress granules in the four-cell stage embryos did not differ significantly among the groups (H_2,61_ = 0.86, *p* > 0.7). However, in the IVF, 35 °C group, there was a significant decrease in the stress granule number at the four-cell stage compared to the two-cell stage (Z = 2.36, *p* = 0.016; Mann–Whitney test). Furthermore, the temperature during the first division influenced the variability of the stress granule number in two-cell embryos (F_2,60_ = 12.39, *p* < 0.000, ANOVA) (Figure 2B). Embryos in the IVF, 35 °C group exhibited significantly greater intra-embryonic variability in the stress granule number (*p* < 0.005, LSD test) compared to the control and IVF, 37 °C groups.

### 2.3. Assessment of Fluctuating Asymmetry of Gene Expression in the Left/Right Forelimbs of Adult Offspring

To evaluate the effect of the incubation temperature during ZGA on the gene expression stability, we randomly selected 10 highly expressed genes (Table 1). Gene expression was measured in offspring at 18 weeks of age in the left and right forelimbs using RT-qPCR. Ct values for each gene were normalized to 18SrRNA, providing estimates of gene expression levels in the left/right forelimbs across the control, IVF 35 °C, and IVF 37 °C groups.

For each offspring group, ΔCt expression matrices containing left/right gene expression data for each embryo were constructed, and eigenvalues were computed. The eigenvalue λ_1_ represents inter-individual fluctuations, while the eigenvalue λ_2_ represents intra-individual fluctuations of gene expression.

**Figure 3:**
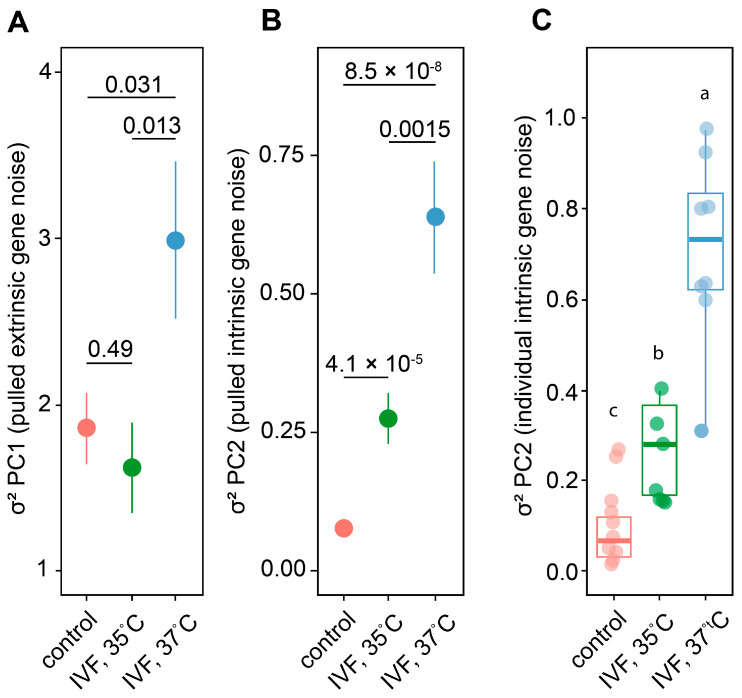
The impact of different temperature IVF conditions on the “extrinsic” (intra-individual) /“intrinsic (inter-individual) (FA)” gene noise in left/right forelimbs. Colors represent different experimental groups: control (red), IVF at 35 °C (green), and IVF at 37 °C (blue). (**A**,**B**) The impact of different temperature IVF conditions on the pulled variance (σ^2^) of “extrinsic (PC1)” and “intrinsic” gene expression noise in the control and experimental (IVF, 35 °C and IVF, 37 °C) groups. Numbers indicate significance level between group differences at FDR < 0.05 for multiple comparisons by *t*-test. (**C**) The impact of different temperature IVF conditions on the individual variance (σ^2^) of “intrinsic” gene noise (PC1) for each animal in the control (n = 6) and experimental IVF, 35 °C (n = 7) and IVF, 37 °C (n = 8) groups. Dots indicate individual variance values of “intrinsic” gene noise (PC2). Letters indicate significant between-group differences at LSD < 0.05 for multiple comparisons by ANOVA test.

Next, the pooled intra-individual and inter-individual fluctuations of gene expression were estimated for each individual. Fluctuating asymmetry in left/right forelimbs assessed by principal component analysis showed that the pooled inter-individual (PC1) and intra-individual (PC2) fluctuating asymmetry values of gene expression were the highest in the IVF 37 °C group (Figure 3A,B). Conversely, the intergroup comparison of mean individual values per animal revealed a significant reduction in expression noise in the IVF 35 °C group (Figure 3C). Therefore, all these gene noise analyses indicate reduced fluctuations in gene expression in offspring incubated at a reduced temperature during the ZGA period.

### 2.4. Hippocampus, Behavior, Metabolites

**Figure 4:**
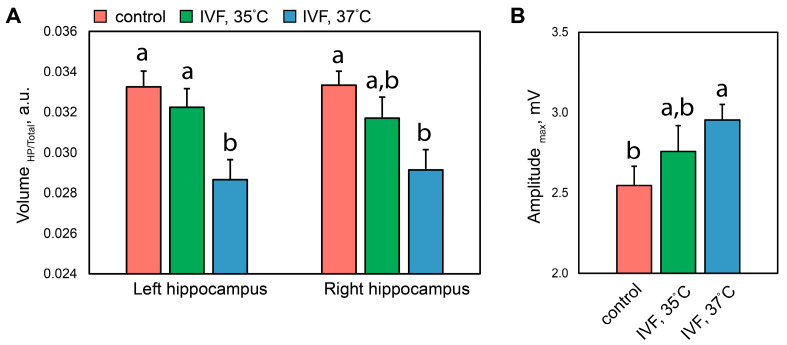
The impact of temperature conditions during the first embryonic cell division on the hippocampus volume and startle reflex of the offspring. Relative hippocampal volume (**A**) and maximum startle reflex amplitude (**B**) in offspring. The data is presented as mean ± standard error: a,b—significant between-group differences (LSD test, *p* < 0.05).

A two-way ANOVA with factors of the offspring group and hippocampal hemisphere (right/left), analyzing relative hippocampal volumes, revealed a significant effect of the experimental group only (F_2,30_ = 10.557, *p* < 0.001). A further post hoc LSD test showed that the relative hippocampal volume was significantly reduced in the IVF 37 °C group compared to both the control group and the IVF 35 °C group on both sides of the hippocampus (Figure 4A).

The groups also differed in behavior in the startle reflex test. Although a one-way ANOVA of the maximum startle reflex amplitude did not reveal significant effects of incubation temperature variation (F_2,30_ = 1.925, *p* = 0.16), a subsequent post hoc LSD analysis showed that the maximum startle reflex amplitude in the IVF 37 °C group was significantly higher than in control animals (*p* < 0.05) (Figure 4B). Additionally, total hippocampal volume values negatively correlated with startle reflex amplitude (r = −0.38, *p* < 0.05).

**Figure 5:**
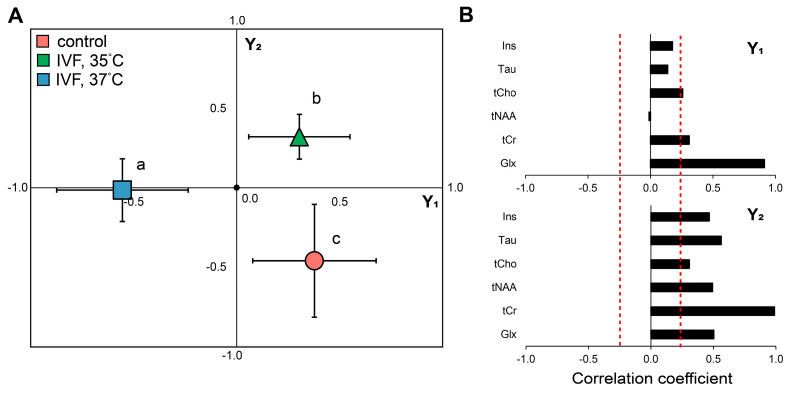
The impact of incubation conditions during the first embryonic cell division on the offspring’s brain metabolism. (**A**) PLS-DA plots of brain metabolites. Group segregation in PLS-DA space (Y1/Y2 axes) following temperature manipulation during initial cell division. (**B**) Correlation coefficients of individual metabolites showing the relationships between neurometabolite concentration changes and the composite metabolic patterns (Y1/Y2 axes) captured PLS-DA dimensions. Data are presented as mean ± SE. Asterisks indicate significant differences from control animals (*p* < 0.05; post-hoc LSD test, one-way ANOVA). Letters indicate significant differences in the IVF, 35 °C group animals (*p* < 0.05; post hoc LSD test, one-way ANOVA) from control and IVF, 37 °C groups. The red dashed line shows the statistical significance threshold for the correlation coefficient (*p* < 0.05).

A two-factor ANOVA analysis, with the factors offspring group and hippocampal hemisphere (right/left), did not reveal a significant effect of these factors on individual metabolite levels. Subsequently, partial least squares discriminant analysis (PLS-DA) of individual neurometabolite levels identified two axes, Y1 and Y2, characterizing variability in the associated variables. The first axis (Y1) positively correlated with the levels of tCho, tCr, and Glx. The second axis (Y2) was positively correlated with Ins, Tau, tCho, tCr, tNAA, and Glx (Figure 5).

One-way ANOVA analysis of Y1 and Y2 axis values revealed a significant effect of embryo incubation temperature (F_2,30_ = 6.826, *p* < 0.005) only for the Y1 axis. For the Y2 axis, the experimental groups did not differ significantly (F_2,30_ = 1.223, *p* = 0.30). Further post hoc analysis showed that the Y1 axis values in the IVF 35 °C group were significantly higher than in the IVF 37 °C group and not significantly lower than in the control group. A significant contribution to the variability of the first axis is made by the concentrations of excitatory neurotransmitters glutamate (Glu) and glutamine (Gln).

## 3. Discussion

There is now substantial evidence that the preimplantation period represents a critical window during which disruptions in the embryonic environment can increase the risk of diseases in offspring [26]. The preimplantation period is characterized by the induction of diverse phenotypes from a single genotype, meaning that individual variations in embryo responses to environmental changes may contribute to the formation of adaptive mechanisms that influence development later in life.

Specifically, we examined responses to acoustic stimuli (startle reflex) in males derived from two-cell embryos that underwent the first division at 35 °C and 37 °C, as well as in a control group. Our results showed that offspring in the 37 °C group exhibited a greater fear response to the acoustic stimulus compared to animals in the control group. Offspring in the 35 °C group did not differ from those in either the control or 37 °C group. Stress response characteristics to such stimuli are associated with risks of psychosis and anxiety disorders, which are modulated by the hippocampus [27]. One possible reason for the increased amplitude of the startle reflex may be alterations in the dentate gyrus of the hippocampus, whose granule cells exert an inhibitory effect on the acoustic startle response, thereby reducing the amplitude of the startle reflex (ASR). Specifically, it has been shown that a single exposure to radiation in newborn rats increases the ASR amplitude and reduces dentate gyrus volume by 60% at five months of age [28]. A comparison of offspring groups exposed to different temperatures during ZGA revealed significant differences in hippocampal volumes. The hippocampal volume on both the right and left sides was lower in the 37 °C group compared to the control and 35 °C groups. Various studies have demonstrated a significant association between type 2 diabetes mellitus and reduced hippocampal volume [29,30,31]. On the other hand, the use of ART may affect lipid and glucose metabolism in children conceived via ART, leading to a high risk of developing type 2 diabetes and metabolic syndrome [32,33,34]. The decrease in the hippocampus size in males in the 37 °C group found in the present study cannot be explained by developmental destabilization caused by disruption of the assembly and disassembly dynamics during SG formation. These data are limited by the time frame of completed cell cycles, and the assembly and disassembly time of the SG ranges from 30 min to 4 h [35,36]. Based on previously obtained data, it can be assumed that developmental disorders of the offspring occur after the second cell division [7]. The increase in inter-blastomere variability in the 5mC level and blastomere sizes of embryos developing in vitro at a constant temperature of 37 °C and a lower number of offspring can be considered as evidence of preimplantation developmental disorders. Overall, this can be considered a possible cause of the observed deviations in the hippocampus.

Metabolomics PLS-DA analysis of offspring group distributions also revealed significant differences in their metabolomics profiles. The 37 °C group differed significantly from the 35 °C and control groups. The main contribution to these differences was from the glutamate–glutamine complex. Glutamate is the dominant excitatory neurotransmitter and the most abundant brain metabolite. It is released during neuronal excitation and converted into glutamine via the glutamate-glutamine cycle, which requires substantial energy expenditure. Changes in their concentrations have been reported in numerous neurological and psychiatric disorders, including depression and mood disorders, epilepsy, and neurodegenerative diseases [37].

Overall, our results demonstrate that embryos exhibit high sensitivity to environmental temperature conditions during the ZGA period. The most pronounced long-term consequences associated with cognitive pathology risks were observed in the offspring of the 37 °C group, which underwent ZGA at temperature conditions typically used in ART procedures. We hypothesize that the observed phenotypic effects are caused by the destabilization of the tightly coordinated developmental program due to suboptimal embryo division temperatures during ZGA.

Fluctuating asymmetry (FA) of bilateral traits is a commonly used measure of developmental instability [13,14,38]. Principal component analysis (PCA) provides a flexible platform for statistically separating “external” and “internal” components of developmental stability [21]. Our analysis of individual PCA1 and PCA2 scores and their means, corresponding to the “external” and “internal” components of transcriptional/developmental stability, revealed destabilization of the transcriptional landscape in the offspring of the 37 °C group. These offspring were incubated during ZGA at temperatures commonly used in ART. In contrast, the transcriptional landscape in the 35 °C group offspring was stabilized. Expression noise in this group was either not different from that of the control group or lower than in the 37 °C group. The stabilization observed in the 35 °C group and the destabilization of the transcriptional landscape in the 37 °C group align well with the phenotypic features of hippocampal function described above. The observed destabilization of the transcriptional landscape in 37 °C offspring, smaller hippocampal volume, and greater response to acoustic stimulation may increase the risk of cognitive impairment. These data suggest that disruption of the embryonic environment during ZGA is associated with long-term consequences that increase expression noise and the risk of pathologies in adult offspring. One of the reasons contributing to the disruption of the developmental program and, as a consequence, destabilization of development in subsequent life history may be heterogeneity of the reaction in individual blastomeres to unfavorable temperature conditions.

Despite morphological homogeneity of murine blastomeres through the eight-cell stage, accumulating evidence demonstrates molecular heterogeneity, characterized by the heterogeneous transcriptional activity of genes governing signaling pathways, transcriptional regulation, and epigenetic modulation [39,40,41,42]. Cells respond to stressors via a range of adaptive mechanisms, such as inducing cell cycle arrest to facilitate damage repair or, under more extreme conditions, activating programmed cell death pathways to mitigate the propagation of cellular injury. Incubation temperature conditions during the ZGA period significantly affect intra-embryonic variability in blastomere size in two- and four-cell embryos and the level of 5mC in blastomere nuclei. This decreases the number of viable eight-cell embryos incubated at 35 °C to levels typical of embryos fertilized in vivo. Meanwhile, intra-embryonic variability of 5mC levels in the 37 °C group markedly increases during this period [6].

One of the earliest cellular responses to adverse environmental conditions is the formation of transient cytoplasmic stress granules (SG), whose life cycle (assembly/disassembly) lasts from 30 min to 4 h [35,36]. SG contains numerous untranslated mRNAs. Their formation serves as a protective mechanism for RNAs; conversely, cells unable to adapt to stress may undergo cell death [36,43]. In the present study, we demonstrated that embryo incubation temperature during ZGA significantly influenced the intensity of stress granule formation. The number of SGs was highest in the 35 °C group during this period. Subsequently, SG numbers decreased to levels observed in the in vivo and 37 °C groups upon increasing the temperature to 37 °C (during the second embryonic division). Interestingly, the peak SG level in the 35 °C group coincided with maximal intra-embryonic (inter-blastomere) variability in SG numbers in two-cell embryos, while the viability of these embryos, assessed by successful progression through the second and third divisions at 37 °C, was minimal. Additionally, lowering the incubation temperature increased the duration of the second embryonic division. We hypothesize that mRNA translation inhibition caused by SG formation and their uneven distribution among blastomeres, combined with delayed early embryonic divisions, creates conditions favoring the elimination of embryos with lower adaptive potential. This finding is corroborated by our prior data demonstrating that embryonic loss rates during pregnancies resulting from two-cell embryo transfers in the 35 °C group were comparable to those observed in the 37 °C group. Moreover, postnatal mortality of neonates in the 35 °C group was lower than in the 37 °C group [6].

**Figure 6:**
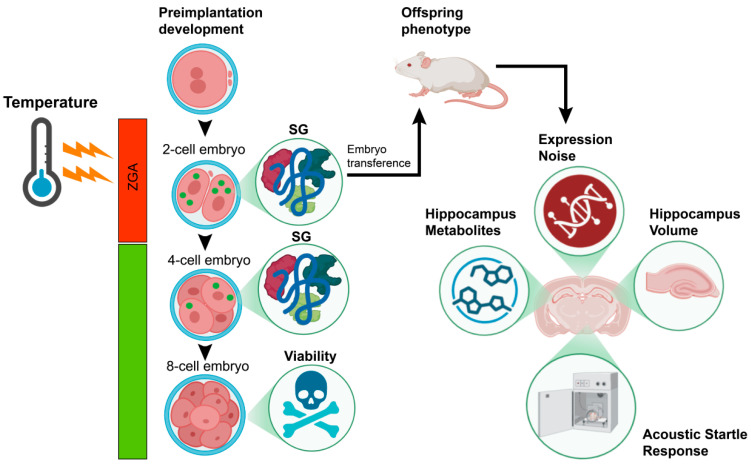
Environmental temperature on the ZGA stage modulates preimplantation development and progeny phenotype.

Thus, embryonic responses to hypothermia were linked to phenotypic traits associated with elevated pathological risk. Expression noise indicative of developmental stability or instability was reduced in the offspring from the 35 °C group compared to those from the 37 °C group, and either did not differ from the control group or exhibited intermediate levels. These findings imply that offspring from the 35 °C group possess enhanced resilience to the adverse effects commonly encountered during embryo transfer procedures. It is also noteworthy that expression noise level, hippocampal volume, and auditory signal responses in offspring that successfully underwent hypothermia during ZGA indicate reduced risks of pathologies related to cognitive functions in this group of offspring (Figure 6).

## 4. Materials and Methods

### 4.1. Animals

The study was conducted on SPF-status outbred CD1 mice aged 14–18 weeks. The animals were housed under a 14-h light/10-h dark photoperiod at a temperature of 22–24 °C and humidity of 40–50%. Food and water were provided ad libitum after autoclaving (121 °C). The animals were kept in individually ventilated cages (Optimice^®^, Centennial, CO, USA: females—5 per cage; males—1 per cage. Animal care and experimental procedures were performed in accordance with the guidelines and recommendations of the Animal Care and Use Committee of the Federal Research Center Institute of Cytology and Genetics, operating under standards set by the Federal Ministry of Health (2010/708n/RF) and the NRC. Experimental protocols were approved by the Bioethics Commission of the Institute of Cytology and Genetics SB RAS (No. 20, 3 November 2022).

Experimental groups:

- Control group: In vivo fertilization, flushing of two-cell embryos, and incubation during the second and third divisions at 37 °C. Offspring obtained via natural mating.
- IVF, 35 °C group: In vitro fertilization (IVF), incubation from zygote to two-cell stage at 35 °C, then transfer to constant 37 °C for the second and third divisions. Offspring obtained by transferring two-cell embryos to recipient females.
- IVF, 37 °C group: IVF, incubation from the zygote to the eight-cell stage at a constant 37 °C. Offspring obtained by transferring two-cell embryos to recipient females.

### 4.2. In Vitro Fertilization (IVF)

IVF was performed using sperm taken from the tail part of the epididymis, which was put in a 100 μL drop of human tubal fluid (HTF) medium covered with mineral oil (Sigma-Aldrich, St. Louis, MO, USA) and kept at 37 °C in 5% CO_2_ for one hour. Virgin CD1 females were superovulated by i.p. injection of 5 IU pregnant mare serum gonadotropin (Folligon, MSD Animal Health, Boxmeer, The Netherlands), followed by 5 IU human chorionic gonadotropin (hCG; Horulon, MSD Animal Health, Boxmeer, The Netherlands) 48 h later. Cumulus–oocyte complexes were collected from the ampulla of the oviduct (15–17 h after hCG injection) and placed in a 200 μL fertilization drop containing HTF. Sperm (3–5 μL from a pre-equilibrated HTF drop) was added to the fertilization drop and incubated for 3–4 h. Fertilized oocytes were washed with four drops of HTF and incubated in 80 μL HTF covered with mineral oil at 35 °C and 37 °C in 5% CO_2_ until the two-cell stage for 24 h. Two-cell embryos were cultured to the eight-cell stage in KSOM AA medium. The duration of cell cycles and embryonic death were monitored using the automated Lionheart FX imager (Biotek, Shoreline, WA, USA) with incubation temperature control up to +40 °C in four chamber zones (4–ZoneTM), as well as condensation, gas environment, and humidity control. The embryos were incubated in 4-well plates (Nunc, Roskilde, Denmark) with 10–12 embryos per 20 µL drop of culture medium. Individual division times and embryonic death were assessed using individual embryo images. For image acquisition, each embryo was brought into focus and individually labeled using the “Add Beacons” function. Embryos that went out of focus were excluded from the analysis. Objectives (Olympus Corporation, Tokyo, Japan) ×20 were used for imaging. The recording interval was 2 h.

### 4.3. Embryo Transfer

The transfer of two-cell embryos was performed as previously described Babochkina et al., 2022 [21]. Two-cell embryos IVF, 35 °C and IVF, 37 °C groups were transferred to pseudopregnant CD1 recipient females obtained by mating with vasectomized males. In total, 199 embryos from the 35 °C group were transferred to 13 females, and 151 embryos from the 37 °C group were transferred to 10 females. The transfers were performed under AErrane gas anesthesia (Baxter Healthcare Corp., Deerfield, IL, USA). Immediately after delivery, the number of newborns and subsequently weaned offspring was counted. After weaning, offspring were separated from their mothers and housed in same-sex groups of five per cage until 18 weeks of age. The control group offspring were obtained from the natural mating of eight females.

### 4.4. Stress Granule Staining

Staining was performed in a sterile Petri dish wrapped in foil. Two- and four-cell embryos were fixed in 10% formalin for 10 min, then washed three times in PBS + 1% TritonX100 (PBST). The embryos were permeabilized with 0.5% Triton-X-100 for 10 min, washed three times in PBS, and blocked with blocking buffer (3% BSA + 1% TritonX100) for 5 min, followed by three PBS washes. The embryos were transferred to a drop containing primary antibodies anti-eIF3b (sc-16377, SCBT, Santa Cruz, CA, USA) and anti-G3BP (611126, BD Transduction, Franklin Lakes, NJ, USA) at a 1:200 dilution and incubated for 1 h at room temperature. After three washes, the embryos were incubated with secondary antibodies anti-goat AlexaFluor 488 (Invitrogen, Carlsbad, CA, USA) and anti-mouse AlexaFluor 555 (Invitrogen) for 1 h at room temperature. Embryo nuclei were stained with 0.01% DAPI for 1 min after three washes. Images were taken with an upright epifluorescence microscope (Axio Imager 2, Carl Zeiss, Oberkochen, Germany) using a 40× objective lens, a laser scanning microscope LSM 780 NLO (Carl Zeiss, Oberkochen, Germany) using a 40× and 100× objective lenses, and an upright epifluorescence microscope (Axio Imager.M2 Colibri 7, Carl Zeiss) using a 40× and 100× objective lenses. Image assembly was performed using Fiji ImageJ 2.15. For experimental validation, we reacted the inhibitor of eIF2α (the α subunit of eukaryotic translation initiation factor 2) and an inhibitor of phosphorylation of PERK kinase assembly of these agglomerates under lower incubation temperature conditions in the IVF, 35 °C group. Stress granule modulators salubrinal (20 μM; SML0951, Sigma-Aldrich, Saint-Louis, MO, USA, an eIF2α dephosphorylation inhibitor) or GSK2606414 (5 μM; Sigma-Aldrich 516535, Saint-Louis, MO, USA, a PERK kinase inhibitor) were added to the culture medium after in vitro fertilization.

### 4.5. Estimation of Extrinsic/Intrinsic Gene Expression Noise

The isolation of total RNA and real-time PCR (RT-PCR) from the left and right forelimbs of mice was based on the guanidine thiocyanate-phenol-chloroform method. The samples were homogenized, and RNA was extracted according to the manufacturer’s protocol (Biolabmix, Novosibirsk, Russia). The concentration of RNA was determined with NanoDrop 2000/2000c (Thermo Fisher Scientific, Waltham, MA, USA). The cDNA was synthesized from 100 mg at 42 °C for 60 min using a random hexamer primer and 100 U of M-MLV reverse transcriptase according to the manufacturer’s instructions (Biolabmix, Novosibirsk, Russia). Briefly, 2 μL of RNA was incubated with 2 μM of a random hexamer primer in a 12 μL final reaction volume at 70 °C for 3 min and then cooled on ice. Then, 16 μL of the reaction mixture containing M-MLV reverse transcriptase (Biolabmix, Novosibirsk, Russia) was added, and the mixture was incubated at 25 °C for 10 min, 42 °C for 60 min, and 70 °C for 10 min. The genes and primers used for RT-qPCR are listed in Table 1. An amount of 2 μL of cDNA samples was added to 20 μL of RT-qPCR SYBR Green master reaction mix containing 0.5 μM of primers. The amplification reaction was carried out on the CFX96 PCR System (BioRad, Hercules, CA, USA). DNA was denatured for 5 min at 95 °C and then amplified in 45 cycles: 95 °C—15 s; 62 °C—50 s. All PCR products were checked by melting curve analysis. RT-qPCR for each sample was duplicated. All genes were evaluated for the measurement error. On average, the standard error of the left/right replicates was 1.52, which is 6.74 times greater than the technical replicate differences.

To estimate the impact of IVF temperature conditions on gene expression noise as an indicator of developmental stability, we randomly selected 10 genes (Table 1). The gene expressions were determined in the left/right forelimbs by RT-qPCR (10 genes) for the animals aged 16–18 weeks in the control (n = 6) group, and experimental IVF, 35 °C (n = 7) and IVF, 37 °C (n = 8) temperature groups. In our previous study [21], we demonstrated that the pooled extrinsic/intrinsic noise of the entire transcriptional landscape could be estimated from a small subset of genes (Table 1) with good to very high accuracy. In the present study, we analyzed the individual and pooled extrinsic/intrinsic gene expression noise in the control and experimental (IVF 35 °C and IVF 37 °C) groups. The extrinsic variance component corresponds to the canalization (L+R). The intrinsic gene expression noise indicates the FA (L−R) since the defining FA is a variance of left/right differences (FA = σ^2^_L−R_) and a particular case of the intrinsic variance component. To remove the effects of directional asymmetry, the extrinsic/intrinsic noise was estimated as eigenvalues of the covariance matrix of left (L)/right (R) gene expressions, where the 1st eigenvalue corresponds to extrinsic noise and the 2nd to the FA [21].

For individual analysis of the FA, the covariance matrices containing left (L)/right (R) gene expressions from an ensemble of 10 genes were constructed for each animal. The 2nd eigenvalue corresponds to an individual FA. For further details of the analysis, refer to Babochkina et al., 2022 [21].

### 4.6. Magnetic Resonance Spectroscopy (MRS) and Imaging (MRI)

All of the studies were performed on an 11.7 T scanner (Bruker, Biospec 117/16 USR, Billerica, MA, USA) equipped with a 9 cm internal diameter gradient (amplitude: 750 mT/m, slew rate: 6660 T/m/s). Hippocampal metabolite levels were studied in groups of males incubated at 35 °C (n = 6), 37 °C (n = 6), and after natural mating (control, n = 6). Metabolite concentrations were determined using the MRS method. Five minutes before analysis, the animals were immobilized under gas anesthesia (Isoflurane, Baxter International Inc., Deerfield, IL, USA) using an inhalation anesthesia apparatus (The Univentor 400 Anaesthesia Unit; Univentor Ltd., Zejtun, Malta). Their body temperature was maintained via a water circuit in the scanner bed with a surface temperature of 30 °C. A pneumatic respiration sensor (SA Instruments Inc., Stony Brook, NY, USA) was placed under the lower torso to monitor anesthesia depth.

Mouse MRIs were acquired using a 1H volume radiofrequency coil (T11440V3). Hippocampal volume was determined using T2-weighted images in axial orientation acquired with the TurboRARE method (Turbo Rapid Acquisition with Relaxation Enhancement). Imaging parameters: pulse sequence (TE = 22 ms, TR = 2500 ms); images sized (2 × 2 cm, matrix 256 × 256 pixels), slice thickness × 0.5 mm, slice gap 0.5 mm, number of slices 20. The total scan time was 7 min. Hippocampal volumes were measured using Fiji ImageJ 2.15 software.

The spectroscopic voxels (1.2 × 2.5 × 2.5 mm^3^) were positioned using T2-weighted TurboRARE images in axial and coronal orientation acquired with the method described above. All proton spectra were acquired using spatially localized single-voxel spectroscopy via the STEAM method (Stimulated Echo Acquisition Mode) with pulse sequence parameters TE = 3 ms, TR = 5 s, NA = 180. Prior to each spectroscopic measurement, magnetic field homogeneity within the selected voxel was adjusted using the FastMap technique [44]. Water signal suppression in spectra was achieved using variable power RF pulses with optimized relaxation delays (VAPOR) [45].

#### MRS Processing

The LCModel v. 6.3 software package was used for processing experimental 1H MRS spectra and quantifying individual metabolites [46]. Metabolites with Cramer-Rao lower bounds (CRLB), representing percentage standard deviation estimates for each metabolite fit, below 20% were considered reliable and included in this study [46]. The following metabolite levels were assessed by LCModel: total N-acetylaspartate and N-acetylaspartylglutamate (tNAA), total glycerophosphocholine and phosphocholine (tCho), total creatine and phosphocreatine (tCr), combined glutamate and glutamine (Glx), N-acetylaspartate (NAA), myo-inositol (Ins), taurine (Tau), and glutamate (Glu).

### 4.7. Startle Reflex

Fear levels were quantified by measuring the startle response amplitude elicited by an acoustic stimulus using an SR-Pilot apparatus (San Diego Instruments Inc., San Diego, CA, USA). The testing chamber (15 × 19 × 25 cm) contained a Plexiglas platform affixed to a piezoelectric transducer, while a ceiling-mounted loudspeaker delivered auditory stimuli. Following a 3 min habituation period under baseline white noise (65 dB), a startle-eliciting acoustic pulse (115 dB, 40 ms) was presented. Concurrently, the piezoelectric transducer was activated, and the resulting signal was processed by the integrated computer system, yielding startle amplitude values in arbitrary units. The experimental protocol consisted of four consecutive acoustic stimuli delivered at 30 s intervals, beginning 3 min after animal placement. The mean startle amplitude across these trials was computed for each subject. The acoustic startle reflex extinction was evaluated based on two key metrics: (1) the peak startle amplitude and (2) the difference in response magnitude between the first and fourth stimulus presentations.

### 4.8. Statistics

The data are shown as the mean ± SE. Comparison of embryo mortality and stress granule counts in experimental groups has been performed by means of the Kruskal–Wallis and Mann–Whitney tests. The mean values of normally distributed features were compared using two-way ANOVA with the least significant difference (LSD) post hoc test. The number of initial variables, characterizing the relative number of brain metabolites, was reduced with a partial least squares discriminant analysis (PLS-DA). The mean values of hippocampal volumes, startle-reflex amplitude, and metabolites were compared to the control using a 2-way analysis of variance (ANOVA) with a post hoc least significant difference (LSD) test. The correlations of the PLS-DA axes with the metabolites and hippocampal volume with the startle-reflex amplitude were analyzed by Pearson’s method.

## 5. Conclusions

To summarize, substantial perturbations of environmental conditions during the first cell division that significantly alter cell cycle duration disrupt the tightly coordinated developmental preimplantation program of embryos. The underlying mechanisms mediating these effects are likely multifactorial. In the present study, division time correlated not only with an increased abundance of stress granules, which are translation inhibitors, but also with an increased intra-embryonic variability of these granules. Conversely, embryos that survived hypothermic exposure demonstrated enhanced adaptive capacity, as reflected by diminished expression noise and decreased amplitude of the response to stressful auditory stimuli. Further investigations in both animal models and human subjects are required to validate these findings. Nonetheless, even if alterations associated with ART are consistently observed, the potential implications for long-term health outcomes should be interpreted with caution and not overestimated.

## Figures and Tables

**Table 1:** Primer sequences for RT-PCR (5′→3′).

Gene	Description	ENSEMBL	Forward	Reverse
*ACTB*	Actin, beta	ENSMUSG00000029580	GGCTGTATTCCCCTCCATCG	CCAGTTGGTAACAATGCCATGT
*ANXA5*	Annexin A5	ENSMUSG00000027712	ATCCTGAACCTGTTGACATCCC	AGTCGTGAGGGCTTCATCATA
*B2M*	Beta-2 microglobulin	ENSMUSG00000060802	TTCTGGTGCTTGTCTCACTGA	CAGTATGTTCGGCTTCCCATTC
*CD36*	CD36 antigen	ENSMUSG00000002944	ATGGGCTGTGATCGGAACTG	GTCTTCCCAATAAGCATGTCTCC
*DCN*	Decorin	ENSMUSG00000019929	TCTTGGGCTGGACCATTTGAA	CATCGGTAGGGGCACATAGA
*GAPDH*	Glyceraldehyde-3-phosphate dehydrogenase	ENSMUSG00000057666	AGGTCGGTGTGAACGGATTTG	TGTAGACCATGTAGTTGAGGTCA
*HDAC2*	Histone deacetylase 2	ENSMUSG00000019777	TGTGCTTGCCATCCTCGAATT	AGCTTCCTCAACACCATCACC
*HSP90AB1*	Heat shock protein 90 alpha, class B member 1	ENSMUSG00000023944	TCAAACAAGGAGATTTTCCTCCG	GCTGTCCAACTTAGAAGGGTC
*POSTN*	Periostin, osteoblast specific factor	ENSMUSG00000027750	GAGGTCTCCAAGGTCACAAAGT	TGTGTCTCCCTGAAGCAGTCT
*RAC1*	RAS-related C3 botulinum substrate 1	ENSMUSG00000001847	GAGACGGAGCTGTTGGTAAAA	ATAGGCCCAGATTCACTGGTT
*RN18S*	18S ribosomal RNA	ENSMUSG00000119584	CTCAACACGGGAAACCTCAC	CGCTCCACCAACTAAGAACG

## Data Availability

The data that support the findings of this study are available from the corresponding author upon reasonable request.

## Supplementary Materials

The following supporting information can be downloaded at: https://www.mdpi.com/article/10.3390/ijms26168060/s1.

## References

[1] Potiris A., Perros P., Drakaki E., Mavrogianni D., Machairiotis N., Sfakianakis A., Karampitsakos T., Vrachnis D., Antonakopoulos N., Panagopoulos P. (2024). Investigating the association of assisted reproduction techniques and adverse perinatal outcomes. J. Clin. Med..

[2] Novakovic B., Lewis S., Halliday J., Kennedy J., Burgner D.P., Czajko A., Kim B., Sexton-Oates A., Juonala M., Hammarberg K. (2019). Assisted reproductive technol ogies are associated with limited epigenetic variation at birth that largely resolves by adulthood. Nat. Commun..

[3] Silva C., Sartorelli E., Castilho A., Satrapa R., Puelker R., Razza E., Ticianelli J., Eduardo H., Loureiro B., Barros C. (2013). Effects of heat stress on development, quality and survival of Bos indicus and Bos taurus embryos produced in vitro. Theriogenology.

[4] de Barros F.R., Paula-Lopes F.F. (2018). Cellular and epigenetic changes induced by heat stress in bovine preimplantation embryos. Mol. Reprod. Dev..

[5] Ramos-Ibeas P., Heras S., Gómez-Redondo I., Planells B., Fernández-González R., Pericuesta E., Laguna-Barraza R., Pérez-Cerezales S., Gutiérrez-Adán A. (2019). Embryo responses to stress induced by assisted reproductive technologies. Mol. Reprod. Dev..

[6] Stanova A., Kontsevaya G., Romashchenko A., Zuev D., Silvanovich E., Moshkin Y., Gerlinskaya L., Moshkin M. (2025). The Temperature of the First Cleavage Impacts Preimplantation Development and Newborn Viability. Int. J. Mol. Sci..

[7] Wek R.C., Anthony T.G., Staschke K.A. (2023). Surviving and adapting to stress: Translational control and the integrated stress response. Antioxid. Redox Signal..

[8] Protter D.S., Parker R. (2016). Principles and properties of stress granules. Trends Cell Biol..

[9] Anderson P., Kedersha N. (2009). Stress granules. Curr. Biol..

[10] Arimoto K., Fukuda H., Imajoh-Ohmi S., Saito H., Takekawa M. (2008). Formation of stress granules inhibits apoptosis by suppressing stress-responsive MAPK pathways. Nat. Cell Biol..

[11] Panas M.D., Ivanov P., Anderson P. (2016). Mechanistic insights into mammalian stress granule dynamics. J. Cell Biol..

[12] Pertoldi C., Kristensen T.N., Andersen D.H., Loeschcke V. (2006). Developmental instability as an estimator of genetic stress. Heredity.

[13] Zakharov V.M., Shadrina E.G., Trofimov I.E. (2020). Fluctuating asymmetry, developmental noise and developmental stability: Future prospects for the population developmental biology approach. Symmetry.

[14] Graham J.H. (2020). Fluctuating asymmetry and developmental instability, a guide to best practice. Symmetry.

[15] Swain P.S., Elowitz M.B., Siggia E.D. (2002). Intrinsic and extrinsic contributions to stochasticity in gene expression. Proc. Natl. Acad. Sci. USA.

[16] Elowitz M.B., Levine A.J., Siggia E.D., Swain P.S. (2002). Stochastic gene expression in a single cell. Science.

[17] Ilan Y. (2023). Constrained disorder principle-based variability is fundamental for biological processes: Beyond biological relativity and physiological regulatory networks. Prog. Biophys. Mol. Biol..

[18] de Jong T.V., Guryev V., Moshkin Y.M. (2021). Estimates of gene ensemble noise highlight critical pathways and predict disease 607 severity in H1N1, COVID-19 and mortality in sepsis patients. Sci. Rep..

[19] Juarez-Carreño S., Morante J., Dominguez M. (2018). Systemic signalling and local effectors in developmental stability, body symmetry, and size. Cell Stress.

[20] Cano-Fernández H., Tissot T., Brun-Usan M., Salazar-Ciudad I. (2025). A mathematical model of development shows that cell division, short-range signaling and self-activating gene networks increase developmental noise while long-range signaling and epithelial stiffness reduce it. Dev. Biol..

[21] Babochkina T.I., Gerlinskaya L.A., Anisimova M.V., Kontsevaya G.V., Feofanova N.A., Stanova A.K., Moshkin M.P., Moshkin Y.M. (2022). Mother–Fetus Immune Cross-Talk Coordinates “Extrinsic”/”Intrinsic” Embryo Gene Expression Noise and Growth Stability. Int. J. Mol. Sci..

[22] Hunter R.H.F. (2012). Temperature gradients in female reproductive tissues. Reprod. Biomed. Online.

[23] Walters E.A., Brown J.L., Krisher R., Voelkel S., Swain J.E. (2020). Impact of a controlled culture temperature gradient on mouse embryo development and morphokinetics. Reprod. Biomed. Online.

[24] Canovas S., Ivanova E., Romar R., García-Martínez S., Soriano-Ubeda C., García-Vázquez F.A., Saadeh H., Andrews S., Kelsey G., Coy P. (2017). DNA methylation and gene expression changes derived from assisted reproductive technologies can be decreased by reproductive fluids. eLife.

[25] Murayama Y., Abe T., Tang Z. (2024). Temperature Dynamics in Early Pregnancy: Implications for Improving In Vitro Fertilization Outcomes. Appl. Sci..

[26] Fleming T.P., Watkins A.J., Velazquez M.A., Mathers J.C., Prentice A.M., Stephenson J., Barker M., Saffery R., Yajnik C.S., Eckert J.J. (2018). Origins of lifetime health around the time of conception: Causes and consequences. Lancet.

[27] Bast T., Feldon J. (2003). Hippocampal modulation of sensorimotor processes. Prog. Neurobiol..

[28] Błaszczyk J., Walasek G., Woźnicka A., Seress L. (1999). Changes of the acoustic startle reflex in rats with radiation-induced hippocampal lesion. Acta Neurobiol. Exp..

[29] Li M., Li Y., Liu Y., Huang H., Leng X., Chen Y., Feng Y., Ma X., Tan X., Liang Y. (2021). Altered hippocampal subfields volumes is associated with memory function in type 2 diabetes mellitus. Front. Neurol..

[30] Monereo-Sánchez J., Jansen J.F., Köhler S., van Boxtel M.P., Backes W.H., Stehouwer C.D., Kroon A.A., Kooman J.P., Schalkwijk C.G., Linden D.E. (2023). The association of prediabetes and type 2 diabetes with hippocampal subfields volume: The Maastricht study. NeuroImage Clin..

[31] Adachi Y., Ota K., Minami I., Yamada T., Watanabe T. (2021). Lower insulin secretion is associated with hippocampal and parahippocampal gyrus atrophy in elderly patients with type 2 diabetes mellitus. J. Diabetes Investig..

[32] Sakka S.D., Loutradis D., Kanaka-Gantenbein C., Margeli A., Papastamataki M., Papassotiriou I., Chrousos G.P. (2010). Absence of insulin resistance and low-grade inflammation despite early metabolic syndrome manifestations in children born after in vitro fertilization. Fertil. Steril..

[33] Ceelen M., van Weissenbruch M.M., Roos J.C., Vermeiden J.P., van Leeuwen F.E., Delemarre-van de Waal H.A. (2007). Body composition in children and adolescents born after in vitro fertilization or spontaneous conception. J. Clin. Endocrinol. Metab..

[34] Belva F., Painter R., Bonduelle M., Roelants M., Devroey P., De Schepper J. (2012). Are ICSI adolescents at risk for increased adiposity?. Hum. Reprod..

[35] Hu S., Zhang Y., Yi Q., Yang C., Liu Y., Bai Y. (2023). Time-resolved proteomic profiling reveals compositional and functional transitions across the stress granule life cycle. Nat Commun.

[36] Klein P., Kallenberger S.M., Roth H., Roth K., Ly-Hartig T.B.N., Magg V., Aleš J., Talemi S.R., Qiang Y., Wolf S. (2022). Temporal control of the integrated stress response by a stochastic molecular switch. Sci. Adv..

[37] Ramadan S., Lin A., Stanwell P. (2013). Glutamate and glutamine: A review of in vivo MRS in the human brain. NMR Biomed..

[38] Klingenberg C.P. (2019). Phenotypic plasticity, developmental instability, and robustness: The concepts and how they are connected. Front. Ecol. Evol..

[39] Roberts R.M., Katayama M., Magnuson S.R., Falduto M.T., Torres K.E. (2011). Transcript profiling of individual twin blastomeres derived by splitting two-cell stage murine embryos. Biol. Reprod..

[40] Biase F.H., Cao X., Zhong S. (2014). Cell fate inclination within 2-cell and 4-cell mouse embryos revealed by single-cell RNA sequencing. Genome Res..

[41] Shi J., Chen Q., Li X., Zheng X., Zhang Y., Qiao J., Tang F., Tao Y., Zhou Q., Duan E. (2015). Dynamic transcriptional symmetry-breaking in pre-implantation mammalian embryo development revealed by single-cell RNA-seq. Development.

[42] Casser E., Israel S., Schlatt S., Nordhoff V., Boiani M. (2018). Retrospective analysis: Reproducibility of interblastomere differences of mRNA expression in 2-cell stage mouse embryos is remarkably poor due to combinatorial mechanisms of blastomere diversification. MHR Basic Sci. Reprod. Med..

[43] Aulas A., Fay M.M., Lyons S.M., Achorn C.A., Kedersha N., Anderson P., Ivanov P. (2017). Stress-specific differences in assembly and composition of stress granules and related foci. J. Cell Sci..

[44] Gruetter R. (1993). Automatic, localized in vivo adjustment of all first and second order shim coils. Magn. Reson. Med..

[45] Tkáč I., Starčuk Z., Choi I.Y., Gruetter R. (1999). In vivo 1H NMR spectroscopy of rat brain at 1 ms echo time. Magn. Reson. Med. Off. J. Int. Soc. Magn. Reson. Med..

[46] Provencher S.W. (2001). Automatic quantitation of localized in vivo 1H spectra with LCModel. NMR Biomed. Int. J. Devoted Dev. Appl. Magn. Reson. Vivo.

