# Supplementary material for: Induction of Stress Granules and Developmental Instability of Offspring Phenotype Due to Hypothermia During First Mouse Embryo Cleavage
Source: Int J Mol Sci. 2025 Aug 20;26(16):8060. doi: 10.3390/ijms26168060 (PMC12386967; doi:10.3390/ijms26168060)
Supplement: Supplementary file 1 [file ijms-26-08060-s001.zip › ijms-3739028-supplementary.pdf]

Supplement Materials

Induction of Stress Granules and Developmental Instability of Offspring Phenotype Due to Hypothermia During First Mouse Embryo Cleavage

Galina Kontsevaya [1], Alexander Romashchenko [1,2], Tatyana Babochkina [1], Dasha Sugatova [1], Oleg Shevelev [1], Marina Sharapova [1], Yuri Moshkin [1,3], Mikhail Moshkin [1,4,*] and Ludmila Gerlinskaya [1,*]

[1] Federal Research Center Institute of Cytology and Genetics, Siberian Branch of RAS, Lavrentyeva 10, 630090 Novosibirsk, Russia; koncevayagalina@bionet.nsc.ru (G.K.); babochkinat@bionet.nsc.ru (T.B.); dasha.sugatova@mail.ru (D.S.); shevelev@bionet.nsc.ru (O.S.); sharapova@bionet.nsc.ru (M.S.); moshkin.yuri@gmail.com (Y.M.)
[2] LIFT Center LLC, 121205 Moscow, Russia
[3] Gene Learning Association, 1205 Geneva, Switzerland
[4] Department of Vertebrate Zoology and Ecology, Tomsk State University, 634050 Tomsk, Russia
* Correspondence: mmp@bionet.nsc.ru (M.M.); lgerlinskaya@gmail.com (L.G.)

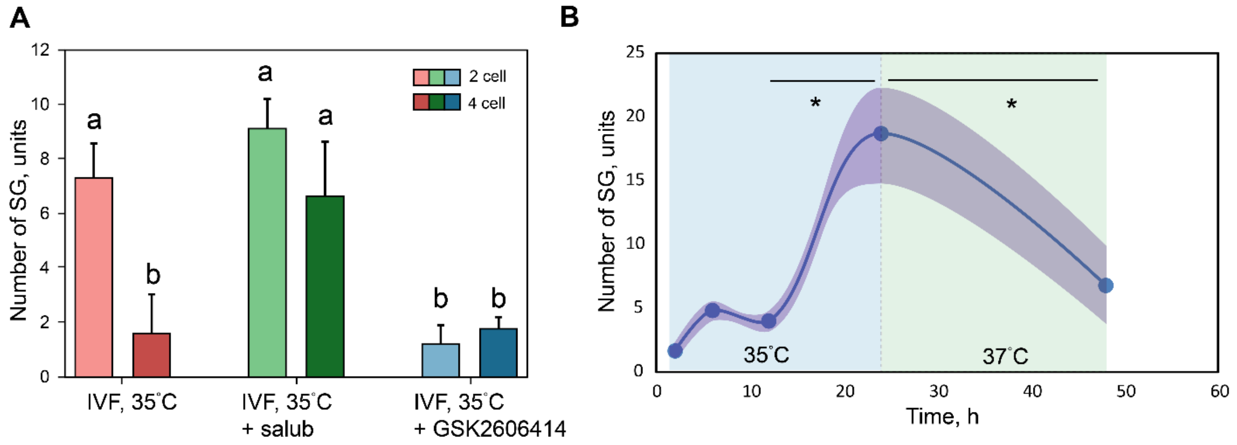

Figure S1. Dynamics (B) and pharmacological modulation (A) of stress granule formation under temperature stress. Following IVF, embryos were kept at 35°C for the first 24 hours before transfer to 37°C. Embryos were treated with salubrinal (20 µM, eIF2α dephosphorylation inhibitor) or GSK2606414 (5 µM, PERK kinase inhibitor) for 48 hours post-IVF under stress conditions. a, b - letters indicate groups that differ significantly (Kruskal- Wallis test $p<0.000$) and pairwise comparisons ($p<0.002$; Mann-Whitney test). * - significant intergroup difference (Students *t*-test, $p<0.05$).
